# Supplementary material for: Hybrid Models and Biological Model Reduction with PyDSTool
Source: PLoS Comput Biol. 2012 Aug 9;8(8):e1002628. doi: 10.1371/journal.pcbi.1002628 (PMC3415397; doi:10.1371/journal.pcbi.1002628)
Supplement: Text S4 — Complete source code for the PyDSTool package (version 0.88.120504). Includes API documentation and help files linking to web pages. This file is identical to the current public release on Sourceforge.net. (ZIP) [file pcbi.1002628.s004.zip › PyDSTool/html/PyDSTool.Toolbox.optimizers.line_search.backtracking_search-pysrc.html]

xml version="1.0" encoding="ascii"?


PyDSTool.Toolbox.optimizers.line\_search.backtracking\_search


| Home | Trees | Indices | Help | PyDSTool |
| --- | --- | --- | --- | --- |

Package PyDSTool ::
Package Toolbox ::
Package optimizers ::
Package line\_search ::
Module backtracking\_search

[hide private]

[frames] | no frames]

# Source Code for Module PyDSTool.Toolbox.optimizers.line\_search.backtracking\_search

```
 1   
 2  # Matthieu Brucher 
 3  # Last Change : 2007-08-26 19:43 
 4   
 5  import numpy 
 6   


7 -class BacktrackingSearch(object):


8    """ 
 9    The backtracking algorithm for enforcing Armijo rule 
10    """ 


11 -  def __init__(self, rho = 0.1, alpha_step = 1., alpha_factor = 0.5, **kwargs):


12      """ 
13      Can have : 
14        - a coefficient for the Armijo rule (rho = 0.1) 
15        - an alpha factor to modulate the step (alpha_step = 1.) 
16        - an alpha factor < 1 that will decrease the step size until the rule is valid (alpha_factor = 0.5) 
17      """ 
18      self.rho = rho 
19      self.stepSize = alpha_step 
20      self.stepFactor = alpha_factor

21   


22 -  def __call__(self, origin, function, state, **kwargs):


23      """ 
24      Tries to find an acceptable candidate 
25      """ 
26      direction = state['direction'] 
27      if 'initial_alpha_step' in state: 
28        alpha = state['initial_alpha_step'] 
29      else: 
30        alpha = self.stepSize 
31   
32      f1temp = function(origin) 
33      gradient = state['gradient'] 
34      while(True): 
35        ftemp = function(origin + alpha * direction) 
36        #Armijo rule 
37        if ftemp <= f1temp + self.rho * alpha * numpy.dot(gradient, direction): 
38          state['alpha_step'] = alpha 
39          return origin + alpha * direction 
40        alpha = alpha * self.stepFactor

41
```

  


| Home | Trees | Indices | Help | PyDSTool |
| --- | --- | --- | --- | --- |

|  |  |
| --- | --- |
| Generated by Epydoc 3.0.1 on Fri May 4 15:24:18 2012 | http://epydoc.sourceforge.net |
